# Mixed tenure communities as a policy instrument for educational outcomes in a deprived urban context?

**DOI:** 10.1080/17535069.2015.1095349

**Published:** 2015-10-15

**Authors:** Oonagh Robison, Ade Kearns, Linsay Gray, Lyndal Bond, Marion Henderson

**Affiliations:** ^a^ MRC CSO Social and Public Health Sciences Unit, University of Glasgow, Glasgow, UK; ^b^ Department of Urban Studies, University of Glasgow, Glasgow, UK; ^c^ Centre of Excellence in Intervention and Prevention Science, Carlton South, Australia

**Keywords:** mixed communities, educational outcomes, neighbourhood context, school context

## Abstract

This article considers mixed community strategies, enacted through planning and regeneration policies, as a policy approach to the improvement of educational outcomes in schools. Analysis is undertaken of educational outcomes across secondary schools in Glasgow. The level of owner occupation in the catchment is positively associated with both examination results at S4 and positive destinations post-school, particularly at the more deprived end of the school spectrum. The results suggest that tenure mix may be both directly and indirectly related to school performance, with neighbourhood context effects not being entirely mediated through the school context.

## 1. Introduction

Children from poorer areas have less positive academic outcomes in terms of both attainment and post-school destinations, and schools in deprived areas tend to have poorer overall outcomes than those in more affluent areas (Teese et al., 2007). In order to overcome this inequality in education, a range of policy approaches has been assembled in advanced societies such as the UK. These include policies to improve the quality of the school estate in deprived areas (Scottish Government & COSLA 2009); boost educational resources; improve the training, recruitment and retention of high-quality teachers (Donaldson 2010; McCormac 2011); and improve school leadership (Education Scotland 2000). Choice in education has also played a role, ostensibly giving all parents the right to select the best school for their child, but also potentially cementing differences between the best and worst performing schools as the parents of more able children avoid the weaker schools (Burgess, Propper, and Wilson 2005). An alternative approach to achieving more mixed (or ‘comprehensive’) schools is to convert more communities to being mixed, in class or income terms, through the use of housing and planning policies and regeneration strategies which pursue ‘inclusive’ or ‘mixed communities’ (Scottish Government 2011a, 2011b). This article assesses whether in theory and in practice such a strategy, pursued through mixed housing tenure developments, could achieve positive impacts upon educational outcomes in secondary schools in a very deprived urban context, such as the city of Glasgow.

In the next section, we consider how two of the key contexts which affect educational outcomes, the neighbourhood and school, operate and interact to have effects within unequal, advanced societies. We then note the rise of mixed tenure housing and regeneration policies in the UK over the past two decades, before reviewing the theoretical and empirical basis for expectations that mixed communities, to be brought about through mixed tenure, can impact upon school performance and educational outcomes. Thereafter, we describe and present the findings from the first stage of an ongoing programme of research looking at the performance of schools and pupils in schools in Glasgow, one of the most deprived cities in the UK, where mixed tenure policies have been underway to restructure erstwhile council housing estates on a large scale. The findings from the school-level data are then discussed in terms of whether or not they provide *prima facie* evidence that mixed tenure strategies for communities might have a positive effect upon educational outcomes in state secondary schools in the city. We also consider whether changes in neighbourhood context can have both direct and indirect – mediated through the school context – effects upon educational outcomes. We end by outlining the direction our future research will take to try to identify the potential mechanisms at work in the neighbourhood-school-context nexus.

## 2. Context effects on educational outcomes

There are two spatial collective contexts which can affect educational outcomes for young people: their neighbourhood and their school, and these may have both independent and linked effects. Variation in these contexts is a much greater possibility in more unequal societies, and studies of literacy have shown that both the differences in educational performance between those at the top and those at the bottom, as well as the average level of achievement, are lower in more unequal societies (Wilkinson and Pickett 2009; Willms 2003; Siddiqi et al. 2007). Wilkinson and Pickett (2009) ascribe these differences in educational performance within unequal societies to a number of factors including the effects of inequality on family relationships, social anxiety and stereotype effects, but also to ‘unequal learning opportunities’ (113).

Support for the ‘unequal learning opportunities’ argument came recently from the OECD’s PISA survey 2012, which reported that ‘striking performance differences [were] also observed between students in advantaged schools and those in disadvantaged schools’ (OECD 2013, 13). Moreover, in the UK case, much more of the difference in performance between schools was explained by the socio-economic intake of students than in most other OECD countries. The UK, along with Belgium, was singled out by the OECD as a country where a high proportion of the difference in performance between schools in the same region, 62% in the case of the UK, was due to the socio-economic status of the school (OECD 2013, 46). Such findings have led to calls for UK policymakers to do more to ensure a better social mix within schools (Glatter 2012). The call for mixed-ability schools and classes in order to raise overall performance has also been made by organisations representing trainee teachers (Paton 2009).

For people from poor backgrounds, often living in poor communities, the educational performance of their local schools matters a great deal since education is a potential route to social mobility (Neelsen 1975). Yet, in affluent countries like Britain, where political support for school selection no longer holds sway, ‘more covert’ methods are employed ‘to divide up groups of children by so-called ability’ so that the performance of local schools is far from equal (Dorling 2010, 33). In a demonstration of the link between the neighbourhood and school contexts, one way this informal selection operates is by parents moving into the catchment area of their chosen school, if they can afford to do so, thus changing the neighbourhood and school contexts at one and the same time.

### 2.1. Neighbourhood effects

The effects upon educational performance of the two contexts of interest here have been more often considered separately than in combination. Research on how a person’s neighbourhood can impact on their lives has been taking place since at least the 1940s, and the belief that neighbourhood has an effect on life chances, including participation and achievement in education and employment, and health outcomes, and that some people are disadvantaged by their neighbourhood (Atkinson and Kintrea 2001) and excluded from taking part fully in society (Forrest and Kearns 2001), is evident in both policy and research. The overwhelming view from the many comprehensive reviews of the existing evidence is that there are small but significant effects of neighbourhoods on individuals, over and above the influence of background characteristics (Jencks and Mayer 1990; Brooks-Gunn et al. 1993; Ellen and Turner 1997; Buck 2001; Sellstrom and Bremberg 2006; Blasius, Friedrichs, and Galster 2007; Galster 2012a).

Neighbourhoods can be seen as part of a social identity, and in this way they are comparative – an individual with adequate resources can use their chosen neighbourhood as a focal point from which to enhance their social positioning (Bridge 2001), while those who are unable to choose their neighbourhoods may find that their social positioning is decided for them (Kearns and Parkinson 2001). The neighbourhood is also an arena in which theories of social capital are played out (Forrest and Kearns 2001): for poorer people, the local community may play a more important social role than for their more affluent counterparts, as for better off residents the neighbourhood is just one of the arenas in which they have ties (Woolcock and Narayan 2000). Therefore, for less affluent residents, the neighbourhood has more often served as an arena for the ‘close-knit and intensive stock of “bonding” social capital that they can leverage to “get by”’ (Woolcock and Narayan 2000: 227) rather than as a platform for the more ‘diffuse and extensive’ (Woolcock and Narayan 2000, 227) ‘bridging’ social capital that enables people to ‘get on’ (Kearns and Parkinson 2001).

Reviews of the evidence of neighbourhood effects on children and young people have found that neighbourhoods with low mean socio-economic status have been associated with negative childhood development, low birth weight, behavioural problems, injury and experiencing child abuse (Sellström and Bremberg 2006). In terms of development and education, high neighbourhood socio-economic status has been associated with adolescent achievement, completing high school, attending college, years of schooling, educational aspiration and occupational aspirations of boys (Garner and Raudenbush 1991; Brooks-Gunn et al. 1993; Duncan 1994; Biggart and Furlong 1996; Furlong and Biggart et al. 1996; Brooks-Gunn and Duncan et al. 1997; Leventhal and Brooks-Gunn 2000).

US work on neighbourhood effects has more often than UK research focused on race alongside the effect of the neighbourhood (Peach 1996) as it can be difficult to disentangle the two. A recent review of educational disparities in the US concluded that whilst educational performance differences largely reflect socio-economic differences between ethnic groups, school factors also play a part (APA Task Force 2012). Ethnic minority students mostly attend schools that continue to be largely segregated to their own ethnic group. The school composition reflects neighbourhood contexts, and where school districts are highly segregated, test scores for students are lower (Vigdor and Ludwig 2008). Even though racial diversity in schools can reduce the educational performance gap, this effect is often reduced due to academic ‘tracking’ within schools (Mickelson 2001).

Although school and neighbourhood segregation by ethnicity is less extreme in the UK than the US (Johnston et al. 2006), nonetheless differences in educational outcomes by ethnic background have been reported in Britain (Gillborn and Mirza 2000). Glasgow, however, has until recently been a predominantly white city. Although Glasgow has had a large and growing Asian community since the mid-twentieth century (McGarrigle and Kearns 2009), the city’s ethnic minority population has traditionally been small and not very diverse for a place of its size. However, over the inter-census period 2001 to 2011, Glasgow’s ethnic minority population more than doubled, from 42,000 (7.2% of the city population) to 92,000 (15.4%), a rate of increase higher than the national average (Freeke 2013). Of the city’s 56 planning neighbourhoods, 5 had an ethnic minority population of 12% or more in 2001, but by 2010 this had risen to 11 neighbourhoods (Freeke 2012a). The two main drivers of these changes have been economic migrants following the enlargement of the European Union, and asylum seekers dispersed to Glasgow under UK Home Office policy. The evidence to date on the impact of migrant children within Glasgow’s schools has shown a positive impact on the social and global awareness of native children and ‘no negative impact on attainment’ (Dillon 2013, 20).

With regard to educational outcomes and the neighbourhood context, the most favoured argument is that having ‘advantaged’ neighbours is the key to any effect due to the benefits offered by their social networks, social norms, role model influence and collective socialisation effects, all of which reinforce positive attitudes to educational attainment, and possibly avoid an alternative focus on antisocial behaviours or diversionary activities (Jencks and Mayer 1990; Sampson, Morenoff, and Gannon-Rowley 2002).

### 2.2. School effects on education

The effect that a school has on pupil outcomes, or the ‘school effect’, can be measured as the variance in outcomes that is unexplained after pupils’ background and prior attainment have been controlled for (Macbeath and Mortimore 2001), and has been found to be between 8% and 15% (Sellström and Bremberg 2006). School effectiveness research seeks to identify ‘best practice’ in schools that are producing good outcomes, with the idea that when these practices are identified and implemented in other schools that they will also see improvements (Rutter and Maughan 2002). Factors such as strong leadership, high levels of parental involvement, an orderly environment and shared sense of mission among staff are among factors to be identified with school effectiveness (Teese and Polesel 2003). In 1997, a review by Scheerens and Bosker identified over 700 factors; however, in more recent research the number has been reduced (Macbeath and Mortimore 2001).

With regard to the school context, a similar argument to that concerning neighbourhood context, about the effects of school composition on collective motivation, attitudes and aspirations, exists (Coleman et al. 1966), but latterly this has been expanded beyond so-called peer group effects to include the mediating effects of composition upon teaching processes, school organisation and school management, that is, who is in the school affects how the school is run (Thrupp, Lauder, and Robinson 2002); this may include the effects of school composition upon the school’s resources, curriculum content and teacher quality (Phillips and Chin 2004). School culture is also important, but that culture is partly a response to the culture of the dominant student group, and in particular their levels of motivation, ability and compliance, that is, schools adapt their processes according to the attitudes of their students, and those attitudes partly reflect where students come from and their assessments of their likely future occupations, illustrating once again a connection between neighbourhood context and school context effects (Phillips and Chin 2004; Thrupp 1999; Rumberger and Palardy 2005).

### 2.3. Evidence for neighbourhood and school effects on education

Over the last decade, a number of studies have examined both neighbourhood context and school context effects upon educational outcomes, though not often testing whether the effects of the two contexts are interactive and multiplicative (Cook 2003). Research in Sweden, Finland and the Netherlands has shown that the effects of neighbourhood socio-economic status on educational outcomes for youth are mediated through the school composition, that is, neighbourhood context effects operate through the school context (Branstrom 2008; Kauppinen 2008; Sykes and Musterd 2011). The Dutch research on secondary school test scores also investigated interaction effects between the two contexts and found no evidence that one context was moderating the effects of the other, concluding that neighbourhood context effects were indirect – operating via changes in the school context – rather than direct. US research also indicates that the effects of neighbourhood context are mediated via the school context, including composition and other characteristics (e.g. Ainsworth 2002), but with two interesting exceptions: in the case of migrant youth, neighbourhood context effects can remain after taking into account school context (Pong and Hao 2007); and neighbourhood racial segregation can still have effects on educational scores after the inclusion of school segregation measures, whilst the latter then has no effect, indicating a substitution of one effect for the other (Card and Rothstein 2006).

Two US studies have investigated interactions between contexts. Cook et al. (2002) found that the four contexts they examined – peer group, family, neighbourhood and school – had additive effects upon students’ grades, attendance record and school activities, but no interactions or multiplicative effects were observed. Owens (2010) examined neighbourhood and school context effects upon high school graduation (possession of a high school diploma) across a national sample of US schools. Neighbourhood concentrated disadvantage – which included measures of poverty, single parenthood and black residents – was a significant predictor of high school graduation, but school composition measures were not. However, there was an interaction between school and neighbourhood contexts in that students from neighbourhoods of low socio-economic status performed worse when in schools with more white and higher socio-economic status pupils. The fact that the relative deprivation of a student’s neighbourhood compared with the neighbourhoods of their student peers had a negative effect upon high school graduation led Owens to conclude that any aim to integrate students from different backgrounds in the same schools should be pursued with care. In other words, the neighbourhood contexts of students and the effects these have upon them should inform the configuration of school contexts if and when calls for more integrated schools are responded to. Elsewhere it has been pointed out that the relationship between neighbourhood context effects and school context effects may differ according to the type of outcome involved be it education, health or antisocial behaviour (Sykes and Musterd 2011; Oberwittler 2007), although a similar point might be made depending on which educational outcome is of concern, be it examination performance or post-school destinations, for instance.

## 3. Housing tenure mix as a policy solution for communities and schools

The development of mixed housing tenure within communities – mixing social rented housing with private sector housing, predominantly owner occupied but also private rented – has been a feature of UK urban policy since at least the early 1990s (Kleinhans 2004; Tunstall 2003), even though it had existed in earlier decades, particularly in the New Town programme of the 1950s and 1960s (Sarkissian 1976; Cole and Goodchild 2001). Unlike the pursuit of broad societal goals of social balance and solidarity as in the post-war New Towns era, the purpose of mixed tenure policies in the recent period has been to avoid problems of concentrated disadvantage and social exclusion in deprived areas as part of strategies for neighbourhood regeneration, and including improving services such as schools for disadvantaged communities (SEU 2001; Berube 2005).

Lupton and Tunstall (2008) note a qualitative change in mixed tenure policy from 2005 onwards, when it became an essential rather than optional part of policy (Tunstall and Lupton 2010), in that the private sector was involved as a much stronger player, and the aim to produce change in a community’s social or population mix was much more explicit than it had been previously, even though the focus on mixed tenure had long been interpreted as a proxy for mixed income (Bailey et al. 2006). As well as mixed tenure forming part and parcel of area-based regeneration programmes, mixed tenure after this date was also to be enacted through a Mixed Communities Initiative for the large-scale renewal of social housing estates (Lupton, Hayden, and Gabriel et al. 2010; Fordham 2009) and also through planning guidelines that required a certain proportion of dwellings in other developments above a minimum size to be ‘affordable’ or socially rented units (Scottish Government 2008a; CLG 2011).

Schools have a central part to play in mixed communities strategies for at least three reasons. First, improving schools in deprived areas is part of the ‘transformative’ attempt to overcome the disadvantage of place, so that people in poor circumstances do not also receive inferior public services but rather have good quality learning environments in mixed schools (Smith and Lupton 2008). Second, new schools are often built as part of a mixed communities project in order to attract and retain middle-income families in the area, offer choice to middle-class households and contribute to neighbourhood stability. Third, by virtue of the fact that ‘as disadvantaged urban communities become more socially mixed, so too will their schools’ (Lupton and Tunstall 2008, 105), mixed communities contribute to achieving a ‘comprehensive ideal’ in education where policies on educational choice may result in ‘increased social stratification’ (Burgess, Propper, and Wilson 2005).

## 4. Theory and evidence for mixed tenure effects on education contexts and outcomes

There are a number of reasons why mixed tenure policies might be expected to have beneficial effects upon the performance of pupils and schools serving mixed communities. However, as we shall see, there is little UK evidence to date for these effects.

Transitions from exclusively council housing to mixed tenure communities can be hypothesised to have beneficial effects upon pupils’ neighbourhood and school contexts for economic, parental/adult and peer group reasons. Owner occupiers are more likely to have jobs and higher incomes than social renters, resulting in improved home, community and school resources for young people, enabling them to study better and live richer lives, thus contributing to educational attainment. There are, however, two qualifications to this argument. First, the differences in income and social background between owners and renters may not be as great as expected within mixed communities (Allen et al. 2005). Second, the local economic and service impacts of more affluent residents is diluted in circumstances where the local services are of modest or poor quality (such as in deprived areas), and owners have greater mobility for consumption elsewhere (Atkinson and Kintrea 2004).

Higher rates of employment among owners are also held to have a potential positive impact upon the aspirations of young people who might observe and experience adults with higher-income or higher-status jobs within mixed communities and/or mix with young people from middle-income households who hold higher aspirations both within neighbourhoods and schools. This assumes, however, that the aspirations of young people from more deprived backgrounds are indeed lower than those of other youngsters, and that career aspirations can be influenced by the employment circumstances of people other than parents and family members, which may not be the case. Indeed, rather than the formation of more ambitious aspirations, a greater challenge may be the ability to access resources to turn aspirations into achievable destinations (St Clair, Kintrea, and Houston 2013).

Relative to estates which are entirely comprised of council housing, mixed tenure communities are expected to provide a more orderly social environment within neighbourhoods and schools, which may assist positive attitudes to learning. This argument stems from two observations about owner occupiers. First, that they exhibit more care and responsibility towards their homes and environments than renters. Second, that owners and higher-income residents tolerate less social disruption and exercise more informal and formal social control than lower-income residents, relating to both their own children and other young people in the area; they try to enforce agreed norms, and more readily contact the authorities when there are problems. Thus, collective efficacy has been shown to increase with the level of owner occupation in an area (Sampson, Raudenbush, and Earls 1997), with the added benefit that informal social control from owners reduces perceived neighbourhood crime and disorder (Lindblad, Manturuk, and Quercia 2013). These effects of having more owners present in a mixed community therefore result in a more orderly environment at home and at school which aids study and reduces the opportunities for young people to get involved in antisocial behaviours which are associated with lower educational attainment. This ‘good neighbour’ effect of responsibility was reported by both professionals and residents (including social renters) in a study of mixed tenure estates in Glasgow (Kearns et al. 2013a, 2013b), whilst lower involvement in crime and antisocial behaviour has been reported for youth who moved from rented housing communities to more mixed neighbourhoods in the US, though the effect was stronger for girls than boys (Ludwig, Hirschfield, and Duncan 2001; Kling, Liebman, and Katz 2007).

There are several major reasons why the theoretical effects of mixed tenure communities upon school and pupil performance may not transpire, relating to both the neighbourhood and school context. Many of the behavioural effects of social or tenure mix within the neighbourhood context depend upon close proximity between owners and renters – for example, so that social observation and cross-tenure informal social control can operate – and upon social interaction between tenure groups (Galster 2007). The spatial configuration of housing tenures within ‘mixed’ communities is rarely considered, but two studies in the UK have reported that residents are more negative about mixed communities in situations where the two main housing tenures are segregated within an estate and are more positively disposed to the other tenure in situations where the tenures are spatially integrated (Silverman, Lupton, and Fenton 2005; Kearns et al. 2013a). The lack of spatial integration between tenures was identified as the biggest barrier to social contact in a study across 10 UK mixed tenure estates (Jupp 1999).

Research in the US and the Netherlands has identified a number of obstacles to social interaction between different income and tenure groups within mixed communities, including the following: it can take time to build up inter-group ties (Clampet-Lundquist 2007); people who move to mixed communities may retain their social ties from their previous neighbourhood (Briggs 1998; Popkin, Harris, and Cunningham 2001); social contact between ‘new’ (more likely owners in the UK case) and ‘old’ (more likely renters) residents may be low because newcomers are externally oriented, reinforced by the low level of amenities within the neighbourhood (Van Beckhoven and Van Kempen 2003). In the UK, researchers have concluded that social interactions between tenure groups are at best ‘superficial’ (Tunstall and Lupton 2010) or ‘civil’, with any cooperative behaviour should it occur being practical rather than personal (Allen et al. 2005). Even where there is close spatial integration between the tenures, it has been found that owners without prior local connections can have ‘minimal’ contact with other residents, whereas renters with local family connections can regard owners with suspicion (Atkinson and Kintrea 2000). One review of the theory and evidence on neighbourhood social mix concluded that ‘… social mix is insufficient to induce substantial social interactions and social capital between groups’ (Galster 2012a), thus casting doubt on whether tenure mix can substantially change the neighbourhood context for young people.

With regard to the school context, the most obvious weak link in the ‘intervention hypothesis’ (Knoepfel et al. 2007) for mixed communities policy is that middle-income, owner occupier parents may not send their children to the local state school. The policy conflict between mixed communities and parental educational choice was identified by Monk, Clarke, and Tang (2011) in a review for the Scottish government. In this case, the change brought about by policy intervention in the neighbourhood context is not transmitted into the school context. One study of a mixed tenure suburb in England showed that owner occupiers and middle-class residents were the most likely to send their children to more distant and better performing secondary schools due to having more information and resources to do so (Camina and Iannone 2014). The authors ‘question[ed] … whether social mix in housing and social mix in education can usefully proceed jointly’ since ‘the more affluent are always in a better position to overcome barriers of cost and time involved in seeking a better school beyond their estate, particularly at secondary level’ (21). In Scotland, the latest figures indicate that the number of placing requests received by local authorities as a share of the S1 school roll (entry stage for secondary school) is 14% across Scotland, but 29% in Glasgow where the research reported here was undertaken (Scottish Government 2010). However, even if the effects of placing requests were absent, it may, however, still be the case that mix within the neighbourhood context is not transferred into mix within the school context for individual pupils due to the ‘institutionalisation’ of social differences that can occur as a result of within-school factors such as subject choice and setting within and between classes (Araujo 2007; Davies et al. 2008).

The latest evidence from the UK government’s recent Mixed Communities Initiative indicates that any effects of developing mixed tenure neighbourhoods upon local schools, that is, the link between changing neighbourhood and school contexts, is uncertain and likely to be delayed, for two reasons. First, the provision or expansion of schools to accommodate a new mixed intake where development is taking place is unlikely to happen prior to the occupancy of new dwellings, and thereafter may be susceptible to delays in housing developments. Thus, although mixed communities have benefited from the provision of new amenities, schools have been the exception (Lupton, Hayden, and Gabriel et al. 2010). Second, the impacts of mixed communities upon local school intakes were often lessened because of the wider catchment area of the school or because the move to higher housing densities (at least in England) favoured the provision of smaller, non-family housing units and because incomers to the mixed developments can be reluctant to change their children’s schools (Fordham 2009). In accord with these findings from the MCI, a recent reassessment of several extant reviews of the UK evidence on mixed tenure concluded that the reviews presented little or no evidence for any positive effects of mixed tenure on local services, including schools (Bond, Sautkina, and Kearns 2011). However, one of the reviews (Holmes 2006) and one of the studies included therein (Silverman, Lupton, and Fenton 2005) indicated a virtuous circle between neighbourhood and school context, wherein a pre-existing school rated as at least ‘good’ could attract patronage from the children of home owners in a new, mixed tenure estate within its catchment.

Apart from the issue of whether neighbourhood context and school context are inter-linked through means of mixed tenure communities, there is also the more fundamental question as to whether or not mixed tenure has effects on educational attainment for pupils or schools. A systematic review of all primary studies in the UK up to 2009 concluded that there was only ‘weak’ and ‘mixed’ evidence for any effects of mixed tenure on educational attainment (Sautkina, Bond, and Kearns 2012), with one study finding a positive effect (Tunstall and Coulter 2006) and another reporting an absence of evidence (Beekman, Lyons, and Scott 2001). This echoes findings from the government’s major area regeneration programme, New Deal for Communities, which included mixed tenure as a policy instrument in deprived areas, that the impacts upon individual outcomes such as educational attainment were very small, if they existed at all, across several indicators (Lawless et al. 2010). All this, despite the fact that improving educational quality (and hopefully also attainment) is an important part of the theory of change for the government’s mixed communities strategy (Tunstall and Lupton 2010). However, as can be seen from the systematic review, the vast majority of research on mixed tenure effects in the UK consists of qualitative case studies, and of the few larger studies using routine data, none had examined educational outcomes (Sautkina, Bond, and Kearns 2012).

## 5. Housing tenure and education in Glasgow

If mixed tenure is to impact significantly on school contexts, with positive consequences for educational performance, then Glasgow is a city where those effects should be evident. Glasgow is a deprived post-industrial city with a relatively poor education record and a substantially changing housing tenure structure.

Glasgow is the most deprived city in Scotland. According to the latest issue of the Scottish Index of Multiple Deprivation (SIMD), 42% of the datazones^1^ in the city lie within the worst 15% in the country, the group which is the target for many public policy interventions. However, Glasgow’s share of the nationally most deprived datazones has been falling in recent years, from 38% in 2004 to 30% in 2012 (Scottish Government 2012b). Glasgow consistently performs worst of all local authorities in Scotland on educational indicators. In 2010/2011, only 25% of S4 pupils in Glasgow City schools received five or more SQA Level 5 qualifications or better compared to the Scotland-wide average of 36%^2^ (Scottish Government 2012b). For the same academic year, 28% of school leavers in Glasgow went on to higher education compared to the Scottish average of 36% (Scottish Government 2011c).

Within the education domain of the SIMD, which uses a combination of pupil and adult measures of educational attainment, Glasgow again has the highest rate of deprivation in Scotland, with 39% of its datazones in the worst 15% grouping for education deprivation, with the next-ranked place being Dundee with a rate of 27% of its datazone in the most deprived group for education. As with deprivation overall, Glasgow’s share of the nationally worst performing areas on education has been falling over time, from 49% in 2004 to 39% in 2012 (Scottish Government 2012b). Looking at aggregate information across local authorities, there are some indications that Glasgow’s underperformance in education may have less to do with education resources than with poverty and its associated cultural and behavioural effects. Pupil teacher ratios in Glasgow, at 13.1, are marginally better than the Scottish average of 13.4 (Scottish Government 2011d). Glasgow has also been heavily investing in its schools estates in recent years, refurbishing and replacing school buildings under the Scottish government’s *Building Better Schools* initiative (Scottish Government 2002; Glasgow City Council 2013). On the other hand, pupil exclusion rates are over six times higher for pupils from the most deprived quintile of areas (many of which as we have seen are in Glasgow) compared with the least deprived (Scottish Government 2011d). The OECD has highlighted the huge gap in educational performance in Scotland, with 71% of pupils from the highest SES backgrounds achieving the benchmark five or more standard grades compared with only 17% of those from the lowest SES group (OECD 2007).

In housing tenure terms, Glasgow has been playing catch-up with the rest of the UK. In 1991, 10 years after the late twentieth century push towards expanding owner occupation across the UK had commenced under the Thatcher governments, Glasgow still had a majority of its households, 57%, living in social rented housing, and owner occupation was a minority tenure at 36% (Freeke 2008). By 2001, owner occupation had reached 49% of all city dwellings. The latest dwelling estimates for the city show that share of households in social renting has reduced to being a minority tenure at 37%, with a diversification of providers such that housing associations are now the largest element within this. Owner occupation has contracted a little over the past decade to 44% of all dwellings, with a shift into private renting, probably due to the economic downturn, so that private renting is now nearly a fifth of all dwellings at 19% (Freeke 2012b).

Tenure mixing within neighbourhoods has also been progressing, such that several of Glasgow’s major council estates, for example, peripheral estates like Castlemilk and Drumchapel, which were almost entirely social rented in 1980, are around a quarter owner occupied today. These changes are as a result of both the Right-to-Buy – with subsequent resales of ex-council houses – and the development of private sector housing on in-fill sites within these large estates. Nonetheless, a recent analysis of housing tenure structures across Glasgow’s datazones reported that one-in-six (16%) were dominated by social rented housing which typically comprised 80–90% of all dwellings within the datazone, whilst one-in-four datazones (24%) now comprised two-thirds social renting and one-third owner occupation (Livingston, Kearns, and Bannister 2013). Thus, we can see that the long-term policy objective to restructure Glasgow’s neighbourhoods to become more mixed tenure has been advancing, but that there is still some way to go. We now turn to the question of whether mixed tenure restructuring could have impacts upon educational outcomes in a poorly performing city.

## 6. Research aims and methods

Our aim was to separately examine the associations of both school context and neighbourhood context with the educational performance of Glasgow’s secondary schools, and then to also examine the association between neighbourhood context and school context. As mentioned previously, we aim to look at whether there is evidence in the first instance that tenure change within a school catchment area could have an impact on educational outcomes. To do this, we have used aggregated data at the school and neighbourhood level.

### 6.1. Data sources and measures used

#### 6.1.1. Neighbourhood context variables

For the measures of neighbourhood context, we construct two variables for the school catchment area. First, we define the school catchments in postcodes and use the Glasgow City Council digitised council tax register for 2008, which includes a record of all occupied dwellings in the city, to measure the proportion of dwellings in each catchment area that are owner occupied, as a measure of housing tenure mix. Second, we use the average rank of the SIMD 2008 for the catchment in which each school is located as a measure of catchment area deprivation.

#### 6.1.2. School context variables

Two measures of school context were used as independent variables, both of which measured the pupil composition in the schools in 2011. The first was the proportion of pupils living in the worst 15% of datazones in Scotland on the SIMD, with a mean value of 55.0% and standard deviation of 22.8%. The second was the proportion of pupils entitled to free school meals: mean 32.5%; standard deviation 11.4%. For a supplementary piece of analysis (see below), we look at two further measures of school context relating to parental choice: the percentage of placing requests into and out of each school for 2012.

#### 6.1.3. Educational performance variables

We used educational performance data for schools provided by Glasgow Education Services for 2011. Glasgow has 38 state-funded secondary schools, including 8 additional support-for-learning schools and 1 Gaelic school; omitting these two special types of school gave us 29 schools for the study.

**Figure 1.**
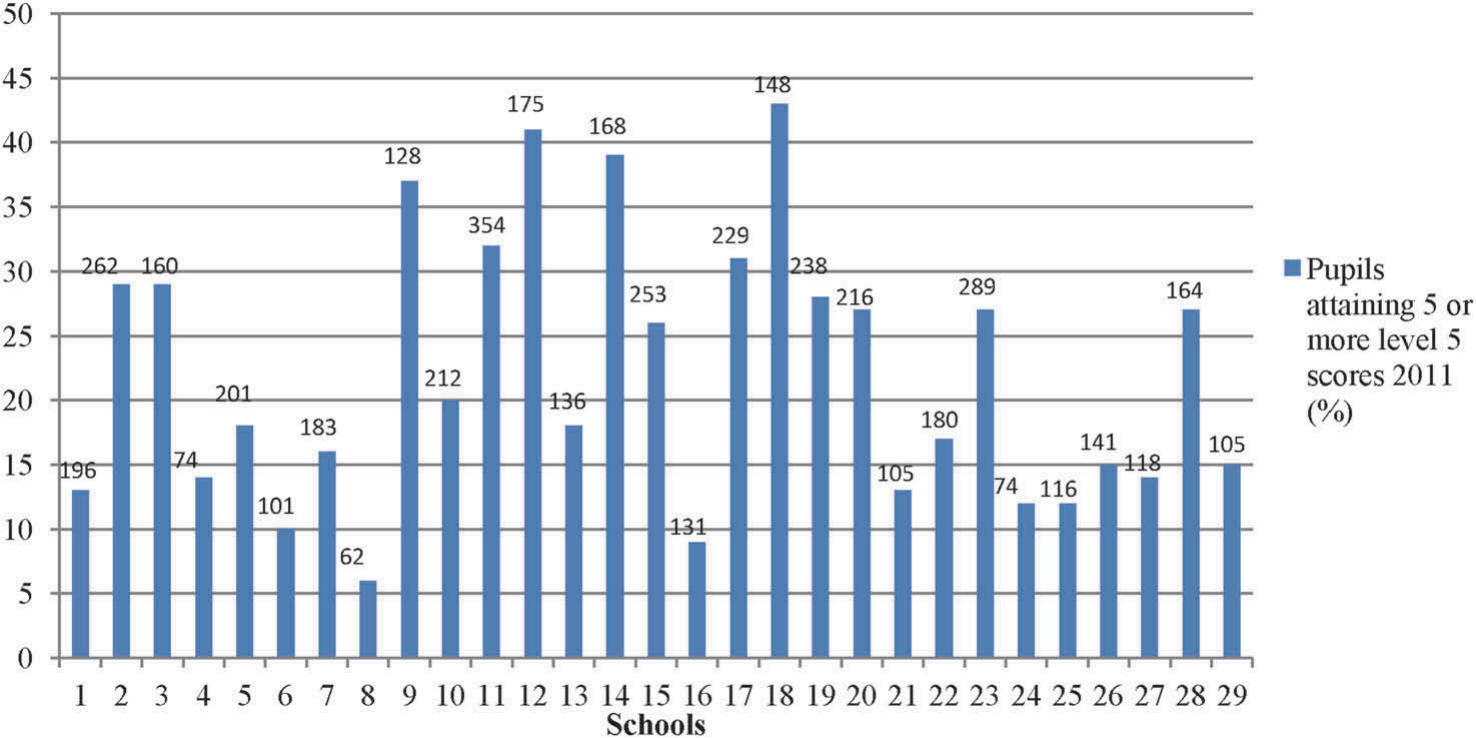
Examination performance across Glasgow secondary schools, 2011. Note: Figures above bars denote *n*, number of pupils in S4 in each school.

Two sets of outcome variables were used to assess different aspects of educational performance, both of which are used in national monitoring. First, we used the number of S4 pupils (aged 15) within each school achieving five or more standard grades at SQA level 5/credit level relative to the total number of S4 pupils. This measure is useful as it captures all pupils as S4 is the last year of compulsory education in Scotland. The measure is also used as an indicator in national monitoring of schools outcomes. However, the measure is relatively blunt as it captures only a binary outcome – either the pupil gained five or more credit standard grades or didn’t – there is no nuance as there would be in using, for example, a composite measure. Figure 1 shows that this measure varied greatly across the schools, from 6% to 43%.

**Figure 2.**
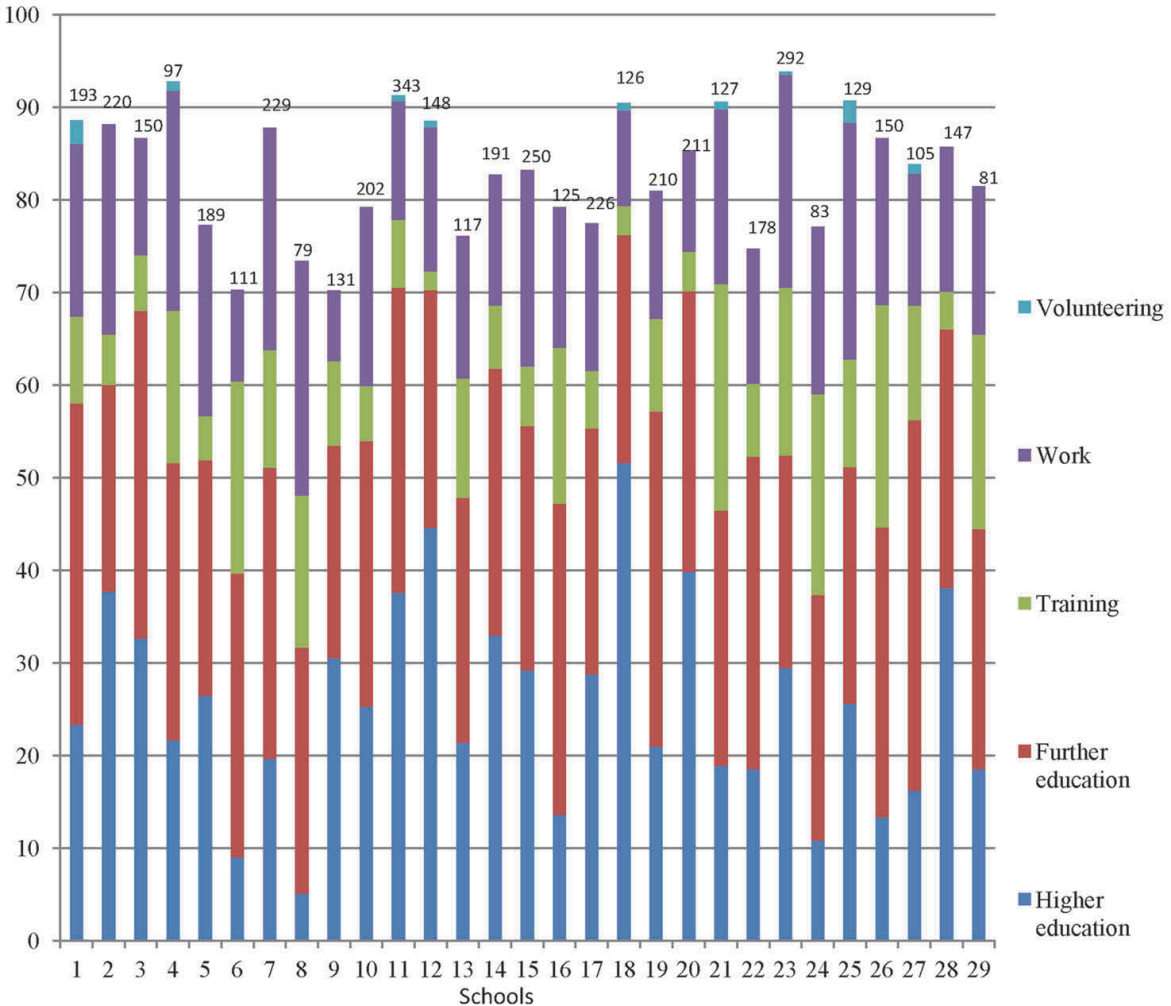
Positive destinations for Glasgow secondary schools, 2011. Note: Figures above bars denote number of school leavers in each school.

The second outcome measure(s) related to the number of school leavers from the 2009/2010 cohort going into positive destinations relative to total leavers, which is a national policy target, and varied from 70% to 93% as shown in Figure 2. It can also be seen that there is a great deal of variation in outcomes for particular types of destination: for example, the proportion of pupils going on to higher education after school varies from 5% to 52%. Thus, as well as using total positive destinations, we also considered three destination subgroups: the proportion of pupils going into work and training, further education and higher education.

### 6.2. Statistical analyses

Firstly, we analyse the five school outcome variables (one for exam results and four for positive destinations) against the two measures of school context/composition. As our data were in counts, as opposed to percentages or proportions, for this we take the standard approach of using negative binomial regression. Though Poisson model regression is the usual method used when analysing count data, due to high variability (‘overdispersion’) in our data, negative binomial was used (Coxe, West, and Aiken 2009). These models provide incident rate ratios (IRRs) which tell us the proportionate difference in the number of pupils within the school achieving each outcome, for a 10% increase in each of the two independent school context/composition variables. Ten per cent was chosen as it was considered to provide results on a meaningful scale for interpretation. The negative binomial regression enables analysis of association with count outcomes whilst taking account of differing overall number of pupils between schools. For the destination-specific analysis, where the type of positive destination is specified, the overall figure is the number of pupils who went on to positive destinations from the school.

Secondly, we analyse the five school outcome variables against the two measures of neighbourhood context using the same modelling approach as above. First, we examine the proportionate difference in the school outcome variables for a 10% increase in the share of owner occupiers in the school catchment. In order to see whether any differential effects operate across the deprivation spectrum in the city, we repeat this analysis, having divided the schools into two groups: the more deprived, being those schools with 40% or more of their pupils from the 15% most deprived areas; and the less deprived being those with fewer pupils than this from deprived areas. Second, we examine the proportionate difference in the outcome variables for a 100 point higher mean SIMD rank (SIMD ranks range from 1 to 6505). Here, higher values correspond to less deprivation within the catchment area, and a change of 100 points was selected to generate meaningfully interpretable results.

Thirdly, we look at the effect of neighbourhood context upon school context by correlating the two measures of school composition against the proportion of owner occupiers in the school catchment. We also correlate the proportion of placing requests into and out of each school against the proportion of owner occupiers within the catchment in order to see whether a higher level of owner occupation – as through the advancement of mixed tenure policies at the lower end of the school/area spectrum – is associated with ‘leakage’ of pupils out of local schools to establishments elsewhere. The rate of approved placing requests into and out of the school was calculated using number of eligible S1 pupils in the catchment as the denominator.

## 7. Results

### 7.1. School context and school performance

Table 1 shows the results for school performance outcomes with school context measures as the independent variables. We can see that the two school context measures are significantly associated with both examination and destination outcomes, though pupils entitled to free school meals have stronger associations than pupils from deprived areas. For a 10% higher number of pupils from deprived areas, the chance of pupils achieving five or more standard grades at credit level was lower by 14.9%. A 10% higher number of pupils receiving free school meals was associated with a 26.3% lower chance of pupils achieving five or more standard grades.

**Table 1.** School context associations with examination results and positive destinations.

	Incident rate ratio (IRR) for educational outcome measure				
School context measure	5+ standard grades at credit level	Overall positive destinations	Higher education	Further education	Work and training
Pupils from deprived areas	0.851**	0.997	0.871*	1.018	1.119**
	[0.821–0.883]	[0.983–1.011]	[0.843–0.899]	[0.989–1.047]	[1.087–1.151]
Pupils on free school meals	0.737**	0.982	0.772**	1.074*	1.152**
	[0.666–0.814]	[0.953–1.012]	[0.696–0.855]	[1.016–1.1135]	[1.053–1.260]
*n*	28^1^	29	29	29	29

Notes: Standard grade results were missing for one school. 95% confidence intervals are in brackets. Ratios represent the effect of a 10% change in the school context measures. Coefficients in bold are significant: * indicates significance at *p* < 0.05; ** indicates significance at *p* < 0.01. ^1^Data were unavailable for one school.

The overall number of pupils moving into positive destinations is not associated with the two school context measures, but there are differential associations with particular destinations. A 10% increase in either school context measure is associated with around 12–15% increases in the chance of pupils going into work or training. A 10% higher number of pupils eligible for free school meals is associated with 7.4% increase in the chance of pupils going into further education. Finally, a 10% increase in pupils from deprived areas is associated with a 12.9% reduction in pupils going into higher education, whilst a 10% increase in pupils on free school meals is associated with a much larger (22.8%) reduction in higher education destinations.

### 7.2. Neighbourhood context and school performance

Table 2 shows that school performance associations are also apparent for neighbourhood context measures and are slightly greater for examination results than for positive destinations. A 10% increase in owner occupiers in the catchment area of a school is associated with a 30.1% increase in the chance of pupils gaining five standard grades at credit level. A 10% increase in owner occupiers is also associated with a 24.1% increase in the chance of pupils going on to higher education and a 13.9% reduction in the chance of pupils going into work or training. An increase in the SIMD rank of 100 is associated with a 3.2% increase in the chance of pupils gaining five or more credit level standard grades. The same increase in SIMD rank has no significant association with the chance of pupils going on to a positive destination overall, but is associated with a 2.7% increase in the chance of pupils going on to higher education, and a 2.2% decrease in the chances of pupils going on to work or training. There is no significant association between an increase in SIMD score and the chance of pupils going on to further education. When the schools are divided into more and less deprived groupings, we see that these associations are present for the more deprived schools (according to pupil intake) but not present for the less deprived schools.

**Table 2.** Neighbourhood context associations with examination results and positive destinations.

	Incident rate ratio (IRR) for outcome measure				
Neighbourhood context measure	5+ standard grades at credit level	Overall positive destinations	Higher education	Further education	Work and training
*Allschools*					
Proportion owner occupiers	1.301**	1.011	1.241**	0.971	0.861**
	[1.194–1.418]	[0.986–1.037]	[1.147–1.344]	[0.923–1.022]	[0.807–0.919]
*n*	28	28	28	28	28
*Moredeprived*					
Proportion owner occupiers	1.313**	0.997	1.260**	0.996	0.867**
	[1.153–1.495]	[0.958–1.039]	[1.112–1.427]	[0.916–1.084]	[0.803–0.936]
*n*	20	20	20	20	20
*Lessdeprived*					
Proportion owner occupiers	1.037	1.027	0.994	0.934	1.098
	[0.921–1.168]	[0.968–1.089]	[0.904–1.092]	[0.840–1.039]	[0.944–1.278]
*n*	8	8	8	8	8
Mean SIMD rank	1.032**	1.000	1.027**	0.996	0.978**
	**[1.022–1.041]**	[0.997–1.003]	**[1.020–1.035]**	[0.990–1.003]	**[0.971–0.984]**

Notes: 95% Confidence intervals are in brackets. All ratios for owner occupiers represent a 10% change. Ratios for SIMD rank represent a 100-point change. Standard grade results unavailable for one school. The more deprived schools are those with 40% or more of pupils from the 15% most deprived areas; the less deprived schools are those with fewer than 40% of pupils from the 15% most deprived areas. Coefficients in bold are significant: ** indicates significance at *p* < 0.01.

### 7.3. Neighbourhood context and school context

Finally, we examined the association between catchment area tenure mix and school pupil composition. There is a significant negative correlation between the proportion of owner occupation in the catchment and the proportion of pupils entitled to free school meals (*r* = −0.67, *p* = <0.01); and a strong negative correlation between the proportion of owner occupation and the proportion of pupils from the 15% most deprived datazones (*r* = −0.87, *p* = <0.01).

**Figure 3.**
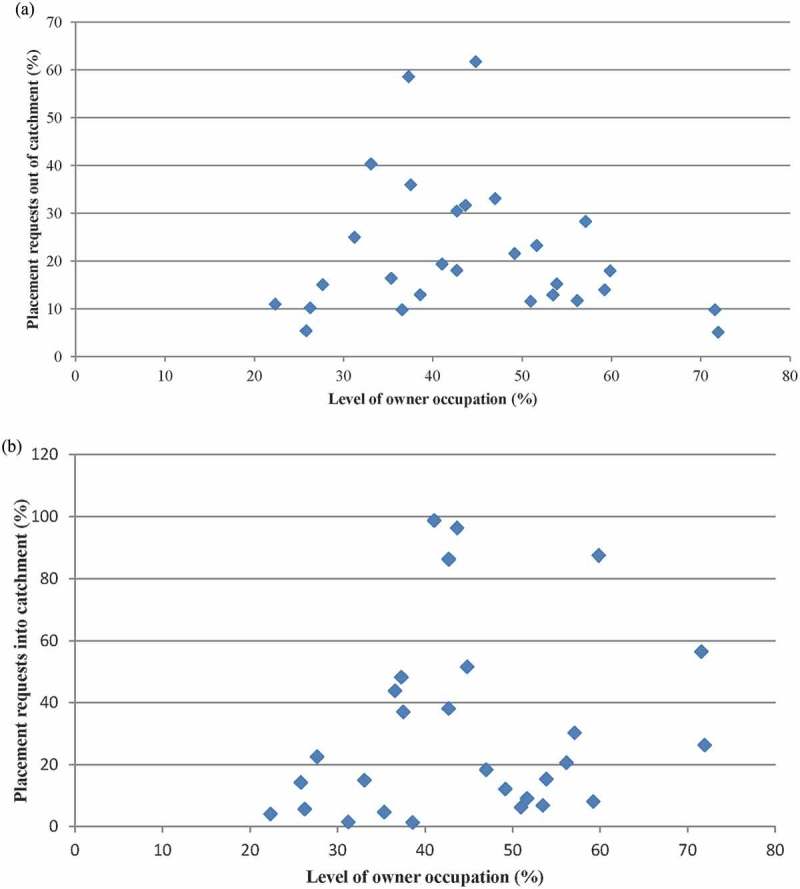
Placement requests at S1 in relation to tenure structure of school catchments. (a) Placement requests out of catchment (%); (b) placement requests into catchment (%). Notes: Figure 3a shows the number of approved placement requests out of a catchment as a proportion of the number of eligible S1 pupils living in the school catchment area. Figure 3b shows the number of approved placement requests into a catchment as a proportion of the number of eligible S1 pupils living in the school catchment area minus the number that have been approved to attend another school. One school has not been included in Figure 3a and b as no calculation of the level of owner occupation in the catchment was available.

We also examined the extent to which owner occupation within the catchment area was associated with approved placing requests into and out of the school. There is no significant correlation between the level of owner occupation in the catchment and the rate of approved placing requests out of a school (*r* = −0.14, *p* = 0.47), nor any significant correlation with the rate of approved placing requests into a school (*r* = 0.19, *p* = 0.33). Scatter plots for both these correlations are shown in Figure 3a and b.

## 8. Discussion

We have examined data for state-funded secondary schools in Glasgow to see if there is any *prima facie* evidence that having more mixed tenure school catchments could feed through into better educational outcomes for pupils. This is an important issue for the city as it has both been pursuing mixed tenure housing, planning and regeneration policies for the last two to three decades (e.g. Glasgow City Council 2004), whilst also languishing at the bottom of education authority league tables over the same period. Our findings show that both the school context (measured in terms of pupil composition) and the neighbourhood context (measured in terms of the tenure structure of the catchment area and average SIMD rank) are associated with educational outcomes within schools, in the direction one would expect. Further, the effects on examination results are slightly greater than upon pupil destinations, particularly for neighbourhood context, although both are affected. We also found strong relationships between the neighbourhood context and school context measures, indicating that the former can affect the latter.

With regard to mixed tenure policy, the level of owner occupation in the school catchment area is associated most strongly with the number of pupils achieving the target number of credit level examination results at age 15 and with progression rates to higher education, both of which can be as low as 10% or less in some schools in Glasgow (Figures 1 and 2). Moreover, the scale of the associations between changes in neighbourhood context and school outcomes is relatively large. Whereas the level of owner occupation in the school catchment neighbourhood is associated with school outcomes for the more deprived schools, we did not find such evidence for the less deprived schools. This set of results is consistent with the notion that neighbourhood context effects are not entirely mediated through the school context, as has been suggested by research in other countries (e.g. Sykes and Musterd 2011).

Scottish education policies are attempting to address disparities in performance. There has since 2003 been an investment programme to improve the quality of school buildings across the country, which has included the refurbishment and new builds of many primary and secondary schools (Scottish Government 2002). This was updated in 2009 as Scotland’s Schools for the Future Programme, with £1.2 billion to be spent on the schools estate up to 2017/2018, with Glasgow City Council publishing its own Schools Estate Strategy to spend £250 million over the 2012–2017 period. National policies for the management of schools, support for pupils with additional needs and plans for the new Curriculum for Excellence are all focused on reducing disparities in educational performance between schools and between pupils from advantaged and disadvantaged backgrounds (Scottish Government 2004). Policies to tackle poverty are also concerned to increase the rate at which school leavers enter positive destinations (Scottish Government 2008b).

The question then arises as to whether policies to create more mixed tenure communities can also make a contribution to educational goals – over and above policies to invest in the school estate, to provide more support for pupils in need and to revise the school curriculum. All this notwithstanding the fact that mixed tenure structures do not guarantee mixed income communities (Musterd and Andersson 2005). These issues are particularly pertinent in Glasgow’s case, given the extent of deprivation, and the fact that examination outcomes vary sevenfold between schools, and destinations such as progressing to higher education vary tenfold, as shown earlier.

Our findings suggest that mixed tenure policies may contribute to improved school performance. However, given that we noted earlier that Glasgow has been pursuing such housing policies for some time whilst continuing to perform relatively poorly in educational terms, it seems the outcomes desired are by no means guaranteed. There are several potential requirements or constraints on mixed tenure effects upon education. First, a lot may depend on who the owners are. US studies that have found associations between mixed communities and the educational performance of poorer youth – for example, in terms of school leaving age (Crane 1991), intellectual functioning scores (Chase-Lansdale et al. 1997) and educational attainment (Duncan, Connell, and Klebanov 1997) – have looked at the percentage of affluent neighbours, usually those in professional and managerial jobs. However, in the case of deprived housing estates in Scotland, it is by no means certain that those who buy homes in such areas will have jobs of much higher status or have very different social backgrounds to others who live in rented housing on those estates. Studies of mixed tenure developments in England have also found that whilst local head teachers wanted a greater representation of professional backgrounds among pupils’ parents the developments tended to attract a limited social range (Allen et al. 2005; Camina and Iannone 2014).

Further, several of the social mechanisms through which mixed communities are expected to impact upon behaviours (including education), such as via role models and through collective social norms, depend upon social contact, both visual and verbal (Joseph 2006) between relatively advantaged and disadvantaged groups, and yet studies have tended to report that such contacts are rare (e.g. Schill 1997; Kleit 2005; Van Beckhoven and Van Kempen 2003). Indeed, research on housing estates in Glasgow has shown that where the different housing tenures are spatially integrated or adjacent (‘segmented’) residents are both more positive about the notion of mixed communities and more likely to report cross-tenure interactions, than where the spatial configuration is a segregated one within the estate (Kearns et al. 2013a). However, other research into the delivery of mixed tenure communities in Scotland has found that the spatial integration of tenures is often resisted by both private developers and social housing providers (Fenton 2010).

There is also the question of how schools and pupils would cope with a more mixed intake, were pupils from owner occupied homes to be of a higher social class grouping than those from rented homes. There is clearly an ongoing debate as to whether, on the one hand, mixed ability classes might lower the performance of the brightest pupils, and, on the other hand, what happens to the self-esteem of less able pupils in mixed ability classes versus classes streamed by ability (Paton 2009, 2012; Ireson, Hallam, and Plewis 2001). Nonetheless, we did not find evidence to support the idea than any impacts of mixed tenure policies are being diluted or lost through the leakage of better-off pupils to other schools due to parental choice – even if it is often a constrained choice (Butler and Hamnett 2010). The relationship between school catchment characteristics and placing requests was modest at best and not statistically significant, and the number of placing requests both in or out of the schools in the bottom third of the spectrum for owner occupation levels in the catchment (i.e. social housing areas with recent private developments) were low (<20 in either direction, per school).

We also consider the reverse effect to be unlikely in Glasgow’s case, whereby higher-income, owner occupiers in mixed tenure developments displace lower-income groups and thus achieve greater dominance in the neighbourhood and school contexts (Lupton and Tunstall 2008). This is for two reasons. First, the kinds of locations subject to mixed tenure policies in Glasgow are not, for the most part, highly valued, high-demand central locations, so that pressure from owners and through market mechanisms is less. Second, Glasgow has a lot of vacant and derelict land, at 7.5% of its total area (Scottish Government 2012c), with 40% of it located within the most deprived areas (double the rate of any other Scottish local authority), and thus there are plenty of available plots and space for new owner-occupied and mixed tenure developments across the city without having to squeeze others out.

### 8.1. Limitations and future research requirements

There are, however, limitations to our analysis and to what we can conclude from it. Ours is not an intervention study, so we cannot say with certainty that the provision of mixed tenure developments within predominantly social housing areas within the city will result in improved performance at local secondary schools, only that in the context we have been examining, it is possible. To verify this, we would need to take advantage of natural experimental conditions whereby we could assess the effects of changing one school catchment but not another (Craig et al. 2012). We also cannot tell through what mechanisms mixed tenure might have effects upon educational performance, nor whether other processes of change within schools also contribute. As our review at the start of this article indicated, there are many possible theoretical routes through which mixed tenure policies might impact upon pupil performance and destinations, operating both within the school and neighbourhood contexts; based on our findings, we think this is likely. However, we aim to conduct qualitative case studies of young people living in different neighbourhood contexts to be able to identify some of these mechanisms in operation – distinguishing, for example, between impacts upon aspirations, positive attitudes, antisocial behaviours and the provision of resources for learning. In addition, school case studies will be conducted to identify some of the impacts of a mixed school context upon school culture and organisation.

Thus, a mixed methods research strategy is appropriate and intended in order to explore context effects on outcomes and in practice. At the same time, research would need to look out for possible negative effects of mixed communities, for example, from competition and relative deprivation between advantaged and disadvantaged neighbours and schoolmates (Galster 2012b), and acknowledge the argument that the benefits of home ownership for child outcomes might stem not from attitudes and behaviours so much as from its ability to provide a wealth cushion and stability of school and home environments (Barker and Miller 2009; Barker 2013; Green and White 1997; Green 2013).

These analyses are based on data available at the school level on a relatively small number of schools. Making use of more detailed data, which we aim to do in the future, would also enable us to address two other major issues in this debate. First, we have yet to measure the very local neighbourhood context around a child’s home, lying at a finer scale than the school catchment area. In a deprived city like Glasgow, local neighbourhoods and gang territories are very real for young people, and it is well known that there no-go areas for some people as a result of micro-belonging (Pickering, Kintrea, and Bannister 2012). Thus, in addition to the catchment area context, there is another crucial scale at which to measure tenure- or social mix, namely the home neighbourhood. Second, there is the question of differential educational impacts from mixed communities, that is, do the most disadvantaged pupils gain from context changes as much as other pupils? Using pupil level data to address this question would also enable us to add a further context or scale to the analysis by placing individual pupils in their classes (be they subject- or setting-based), within their schools. This would allow us to examine whether changes in the neighbourhood context feed through to the school context and into the class context within which individual pupils from different backgrounds conduct their learning.

## 9. Conclusion

Through an examination of circumstances in Glasgow, we have shown that there is at least *prima facie* evidence that mixed tenure policies *could* have positive impacts upon educational outcomes in secondary schools, within a very deprived urban context. We cannot tell why this is the case precisely, although our evidence suggests that the effects of neighbourhood context are likely to operate both outside and inside schools, without necessarily being entirely mediated by the school context. Our aim is to conduct further, more detailed research using individual pupil records across entire school careers to seek to identify the potential mechanisms at work, both positive and negative, as mixed tenure situations arise or endure over time. In doing this, we accept that mixed community policies might be considered no more than ameliorative (though potentially important nonetheless) since ‘they fail to address root causes of poverty and unequal opportunity to learn’ (Lipman 2008, 119). However, in our view, whilst context is not everything, it looks like it might be significant, and therefore, worth further consideration.

## Notes

1. Datazones are the official statistical unit used in Scotland. On average, datazones have a population of between 500 and 1000 people, and there are 694 datazones in Glasgow. The SIMD ranks the country’s 6505 datazones, with 1 being the most deprived and 6505 being the least deprived (Scottish Government 2012a).

2. SQA level 5 national qualifications include standard grades mostly taken in the fourth year of secondary school (SQA 2006).
